# Influence of Information and Communication Technologies on the Resilience and Coping of Sexual and Gender Minority Youth in the United States and Canada (Project #Queery): Mixed Methods Survey

**DOI:** 10.2196/resprot.8397

**Published:** 2017-09-28

**Authors:** Shelley L Craig, Lauren B McInroy, Sandra A D'Souza, Ashley Austin, Lance T McCready, Andrew D Eaton, Leslie R Shade, M Alex Wagaman

**Affiliations:** ^1^ Factor-Inwentash Faculty of Social Work University of Toronto Toronto, ON Canada; ^2^ Dalla Lana School of Public Health University of Toronto Toronto, Ontario, ON Canada; ^3^ School of Social Work Barry University Miami Shores, FL United States; ^4^ Ontario Institute for Studies in Education University of Toronto Toronto, Ontario, ON Canada; ^5^ Faculty of Information University of Toronto Toronto, Ontario, ON Canada; ^6^ School of Social Work Virginia Commonwealth University Richmond, VA United States

**Keywords:** mixed methods, survey, grounded theory, sexuality, LGBTQ, gender, sexual orientation, gay, transgender, youth, Internet, online, information and communication technologies

## Abstract

**Background:**

Sexual and gender minority youth are a population in peril, exemplified by their disproportionate risk of negative experiences and outcomes. Sexual and gender minority youth may be particularly active users of information and communication technologies (ICTs), and it is important to identify the potential contributions of ICTs to their resilience and well-being.

**Objective:**

Our aim was to (1) investigate the use of ICTs by sexual and gender minority youth, (2) identify the ways that ICTs influence the resilience and coping of sexual and gender minority youth, focusing on promotion of well-being through self-guided support-seeking (particularly using mobile devices), (3) develop a contextually relevant theoretical conceptualization of resilience incorporating minority stress and ecological approaches, (4) generate best practices and materials that are accessible to multiple interested groups, and (5) identify whether video narratives are a viable alternative to collect qualitative responses in Web-based surveys for youth.

**Methods:**

Mixed methods, cross-sectional data (N=6309) were collected via a Web-based survey from across the United States and Canada from March-July 2016. The sample was generated using a multipronged, targeted recruitment approach using Web-based strategies and consists of self-identified English-speaking sexual and gender minority youth aged 14-29 with technological literacy sufficient to complete the Web-based survey. The survey was divided into eight sections: (1) essential demographics, (2) ICT usage, (3) health and mental health, (4) coping and resilience, (5) sexual and gender minority youth identities and engagement, (6) fandom communities, (7) nonessential demographics, and (8) a video submission (optional, n=108). The option of a 3-5 minute video submission represents a new research innovation in Web-based survey research.

**Results:**

Data collection is complete (N=6309), and analyses are ongoing. Proposed analyses include (1) structural equation modeling of quantitative data, (2) grounded theory analysis of qualitative data, and (3) an integrative, mixed methods analysis using a data transformation design. Theoretical and methodological triangulation of analyses integrates an interwoven pattern of results into a comprehensive picture of a phenomenon. Results will be reported in 2017 and 2018.

**Conclusions:**

This research study will provide critical insights into the emerging use of ICTs by sexual and gender minority youth and identify intervention strategies to improve their well-being and reduce risks encountered by this vulnerable population. Implications for practice, research, and knowledge translation are provided.

## Introduction

### Information and Communication Technologies

Information and communication technologies (ICTs) are offline (eg, televisions, phones) and online (eg, Internet, social media) technologies that facilitate communication and sharing of information, including mobile devices (eg, mobile phones, tablets) [1]. ICTs have the potential to promote resilience and coping among youth [2]. In the United States, 92% of adolescents (age 13-17) are online daily [3], and 97% of young adults (age 18-29) use the Internet [4]. In Canada, 99% of youth (age 16-24) use the Internet [5]. Global expansion in ICT use is expected to lead to improved education and economic opportunities for youth [6], yet whether this is true of socially marginalized populations remains unknown. The rapid uptake of ICTs represents a promising evolution for accessible interventions [7]. Youth in crisis may turn to online ICTs for support before interventions such as hotlines or social services [8]. There remains a lack of research on the potential positive impacts of increased ICT usage by sexual and gender minority youth.

### Sexual and Gender Minority Youth

Sexual and gender minority youth are youth who identify as gay, lesbian, queer, bisexual, transgender, and other nonmajority sexual and gender identities. They are a population in peril, as exemplified by disproportionate risk of negative experiences and outcomes, such as familial rejection [9], social exclusion [10], depression [11,12], and low academic achievement [13,14]. Such risks result in accumulation of high overall levels of stress [15]. Sexual and gender minority youth are also more likely to endure emotional and behavioral stressors (eg, isolation, violence, victimization) [16-19]. Such experiences exacerbate vulnerability to negative outcomes. Opportunities to foster resilience and coping are critical to sexual and gender minority youth health and well-being. Resilience is defined as the ability to adapt constructively to risk exposure [20], while coping refers to dynamic and conscious actions to regulate behavior in the face of stress [21].

Sexual and gender minority youth are particularly active Internet users compared to their non-sexual and gender minority youth peers [22], often relying on ICTs for sexual and gender minority information and resources [23,24]. There are risks related to ICT use for sexual and gender minority youth, such as increased likelihood of online bullying and harassment [22]. However, there are potential beneficial contributions of these technologies to sexual and gender minority youth resilience and coping that are important to understand. The use of ICTs to engage in self-guided support seeking (ie, using ICTs to independently address one’s own challenges) requires further investigation.

### Study Objectives

This study, Project #Queery, will (1) investigate the use of ICTs by sexual and gender minority youth, (2) identify ways that ICTs influence the resilience and coping of sexual and gender minority youth, focusing on promotion of well-being through self-guided support-seeking (particularly using mobile devices), (3) develop a contextually relevant theoretical conceptualization of resilience incorporating minority stress and ecological approaches, and (4) generate best practices and materials that are accessible to various stakeholder groups (eg, youth, service providers, policy makers). Additionally, using an innovative data collection design, this study will (5) identify whether video narratives are a viable alternative to collect qualitative responses in Web-based surveys for youth. A Web-based survey, open from March-July 2016, generated a sample of 6309 English-speaking sexual and gender minority youth participants aged 14-29 from across the United States and Canada. Data are currently being cleaned and analyzed. The purpose of this paper is to detail the research protocol of Project #Queery, including study design, recruitment strategies, data collection procedures, proposed mixed methods and integrative analyses, as well as planned knowledge mobilization activities.

## Methods

### Study Team

The Project #Queery Study Team consists of (1) a principal investigator, (2) two co-investigators, (3) a research coordinator, and (4) a multidisciplinary advisory group. The advisory group included the co-investigators, two international academic collaborators, several social service practitioners working with sexual and gender minority youth, and a number of sexual and gender minority youth. The advisory group provided insight during measure development, tested the data collection tools, and will provide additional support throughout analyses, dissemination, and knowledge mobilization. The principal investigator and the research coordinator managed the advisory group, as well as a research team of 5-7 research assistants, with ongoing input from the co-investigators. Research assistants were students from all degree levels (ie, bachelors, masters, and doctoral) and were selected for their research skills or knowledge of the target population. Research assistants completed a variety of tasks, including outreach and recruitment, measure development, data collection and management, website and social media development and maintenance, literature searches and reviews, data cleaning and mixed methods analyses, and dissemination activities.

### Ethics and Consent

Prior to completing the Web-based survey all participants, regardless of age, read and independently accepted a Web-based statement of informed consent. Parental consent was not required. Participants who opted to submit a video at the end of the survey read and accepted an additional statement of informed consent prior to completing that section. Obtaining independent informed consent from participants under age 18 may pose challenges [25]. This approach may be justified in specific situations, such as where seeking parental consent may put youth at risk. In the case of Project #Queery, requiring parental consent would have potentially put some participants at risk of exposure regarding their sexual and gender minority youth status [26,27]. The University of Toronto Research Ethics Board approved the protocol for this study, including approving the independent consent procedure for participants under age 18 (ie, waiving the parental consent requirement). Consent materials should be age-appropriate and easy to understand [26]. In Project #Queery, all consent materials were assessed for grade-level readability (using Readable.io) to ensure they were understandable to the youngest participants. Additionally, animated videos explaining each consent statement (ie, survey and video) were provided. Videos were produced using online video animation platform GoAnimate and were implemented to increase participants’ understanding of the study [28].

All standard elements were included in the Web-based informed consent, including an introduction to the study and the estimated time required to complete the survey. Participants were not required to provide identifiable details (other than an email address). Research assistants were available by email, as well as via study accounts on Facebook, Instagram, Twitter, Tumblr, Reddit, and YouTube. Platforms permitted direct/private messaging. National sexual and gender minority youth resources in both the United States and Canada were listed at the end of the survey, and contact information for the principal investigator was provided for more personalized resources. Participants were also asked if they needed immediate help directly after answering challenging questions (eg, self-harm, suicidal ideation) and were provided with immediate access to the aforementioned national sexual and gender minority youth resources [26].

### Data Collection

A Web-based, cross-sectional open survey of sexual and gender minority youth was employed containing a combination of quantitative measures and qualitative questions, as well as enabling the opportunity to provide a video response. The study logo (Project #Queery) and institutional logos (Factor-Inwentash Faculty of Social Work, University of Toronto) were displayed. This research used digital delivery because (1) sexual and gender minority youth are avid users of ICTs, so it is a naturally occurring location for intervention [2,22], (2) youth prefer this approach [29], (3) Web-based research is cost effective [26], and (4) sexual and gender minority youth not present in other systems of care may be captured [30]. Only highly secure and encrypted data collection platforms were used (ie, Qualtrics, WeTransfer). Since data collection was completed, data have been encrypted and kept on secure computer drives. The survey was tested for usability and functionality, including vetting by the advisory group.

Following participation, sexual and gender minority youth had the option to enter into a raffle for a variety of prizes. These include e-gift cards to Amazon or iTunes (100 cards at Can $25, two cards at Can $250), as well as five iPad Minis. Participants were required to provide only an email address to enter the raffle. The number of raffle entries participants received depended on the completeness of their data. Additional prizes were provided for completing the optional fandom and video sections to encourage participation. Fandom refers to communities of individuals with personal connections to particular media objects. Participants who completed a survey received one raffle entry. Participants who also completed a video received a second raffle entry (and entry into a specific draw for an iPad Mini just for video participants). Fandom participants were entered into a specific draw for a Can $100 e-gift card. Raffle winners were selected using a random number table; all prizes were distributed online.

#### Recruitment

A multipronged, targeted recruitment approach using Web-based, purposive, venue-based strategies was used to recruit a diverse, convenience sample of sexual and gender minority youth:

e-Flyers and participation emails were distributed to agencies and organizations serving sexual and gender minority youth in every state in the United States, and every province and territory in Canada. Over 950 groups were contacted (most multiple times).

Approximately 70 Facebook groups (eg, regional Pride pages, pages for campus groups) were directly messaged encouraging them to ask their communities to participate. Communities were also contacted on other social media platforms (eg, Tumblr, Reddit).

Promoted (paid) posts were employed using Facebook’s Ad Manager to reach approximately 98,000 people on Facebook and Instagram. Several of these posts targeted particular locations and communities in an effort to generate a geographically broad and diverse sample [27].

An animated YouTube commercial, produced using GoAnimate, was released. The process of developing and using animated videos in research with youth is described in detail elsewhere [28]. As random sampling is not feasible in hard-to-reach, stigmatized populations [31], existing relationships between the research team and organizations serving sexual and gender minority youth helped identify appropriate recruitment pathways. Limited paper flyering was completed in Ontario, Canada.

#### Survey

Participants (N=6309) completed a 30-45 minute Web-based survey hosted on Qualtrics, a method often used in youth research [26]. Web-based data collection is preferred by adolescents and has been suggested for effectively accessing marginalized populations, including sexual and gender minority youth [26,29,32]. The survey was divided into eight sections: (1) essential demographics, (2) ICT usage, (3) health and mental health, (4) coping and resilience, (5) sexual and gender minority youth identities and engagement, (6) fandom communities, (7) nonessential demographics, and (8) a video submission (optional). Adaptive questioning (where survey items are only conditionally displayed) [33] was used. A “back button” was available to allow participants to revise their answers. Participants were also able to return to their survey and make changes or complete additional survey items for up to 1 week. Unique site visitation was determined via the provided email address, as Internet protocol addresses were not collected [33]. Each page contained a maximum of five questions. The questionnaire was a maximum of 40 pages, provided that participants viewed every question. The retention rate through to the last section of the survey (nonessential demographics) was 76.22% (4809/6309).

#### Measures

Measures incorporated into various sections of the survey included:

1. Demographics (Sections 1 and 7): Developed for Project #Queery, Section 1 contained essential demographics, including measures of age, gender identity, sexual orientation, race and ethnicity, current geographic location (eg, country, state/province), and education attainment (eg, high school, college, university) and educational status (eg, student, nonstudent). Section 7 contained demographics that were deemed important, but less essential to the core research questions, including community type (eg, city, town, rural), employment status (eg, part-time, full-time), country of birth, duration in country of residence, socioeconomic status (eg, parent occupation), living situation, and family religiosity.
2. ICT Usage (Section 2): This was created specifically for Project #Queery and informed by the format and content of the Pew Internet & American Life child and adolescent ICT surveys. Datasets are available on the Pew website. Questions included active hours online per day, devices used and their capabilities (eg, phone, text, WiFi, data), and favorite social media sites and their impact.
3. Identity Development (Section 5): Generated for Project #Queery from a review of the literature on sexual and gender minority youth identity development [34-37], this section included questions on sexual attraction and relationship experiences, ages of realization and identification of sexual and gender minority status, outness and age(s) of coming out, and experiences in sexual and gender minority communities.
4. Fandom Communities (Section 6): Fandom refers to communities composed of individual fans with personal connections to various media objects (eg, television shows, movies franchises) [38,39]. This section asked about participation in fandom communities in online contexts.

Existing scale survey measures were also incorporated (see Table 1 [40-52]).

#### Video

Participants were given the opportunity to enter an open-ended textual response or upload a 3-5 minute video or audio recording in which they provide an example of how ICTs have facilitated their resilience and coping. This research innovation, suggested by sexual and gender minority youth in a previous study as an alternative to open-ended questions (often a part of adolescent surveys), represents an innovative multimedia approach to survey research. Participants who opted to provide a video (n=108) submitted files via WeTransfer, a Web-based file sending service that allows for the free encrypted transfer of files as large as 2GB. The paid version of the service (a “Plus Channel”) provided a personalized webpage where participants were directed to upload their files. All participants were required to provide (in addition to the video file) was a valid email address. The email address allowed the connection of participants’ videos to survey responses and raffle entries. Participants on mobile devices were able to upload files in any mobile Web browser.

**Table 1:** Scale survey measures.

Scale	Survey section	Items, n	Modifications
E-Health Literacy Scale [40]	3	3	5 items excluded
Diagnostic and Statistical Manual of Mental Disorders, V [41]	3	21	(1) Excluded 6 items from sections 9 (Psychosis) and 10 (Repetitive Thoughts and Behaviors). (2) Changed scale from 5-point (0-4) to 11-point (0-10).
Perceived Stress Scale [42,43]	4	10	No modifications
Adverse Childhood Experiences Scale [44]	3	10	Added an unsure response option
Internalized Queerness [45]	4	5	Replaced gay or homosexual with the word queer
Microaggressions [46]	4	9	From LGBQ Microaggressions on Campus Scale Used 5/15 items from interpersonal subscale and 3/5 items from environmental subscale
Child and Youth Resilience Measure-12 [47]	4	12	No modifications
Adolescent Coping Orientation for Problem Experiences [48,49]	4	20	34 items excluded
School Performance and Engagement [14,50]	4	5	From the Youth Risk Behavior Survey
Social Support Scales [51,52]	4	10	Used 5/9 items From Family Cohesion Scale and 5/9 items from Peer Support Scale

**Table 2:** Sexual orientations and gender identities (N=6309).

			n		%
**Sexual orientations**					
	Pan umbrella^a^	1878		29.8	
	Bi umbrella^b^	1655		26.2	
	Queer	1321		20.9	
	Gay	988		15.7	
	Lesbian	983		15.6	
	Asexual (Ace) umbrella^c^	748		11.9	
	Not sure/Questioning	399		6.3	
	Other	153		2.4	
	Demi umbrella^d^	128		2.0	
	Straight/Heterosexual	122		1.9	
	Two-spirit	74		1.2	
**Gender identities**					
	Woman/Female	2592		41.1	
	Gender nonbinary/Nonconforming/Independent	1506		23.9	
	GenderQueer/GenderFluid	1230		19.5	
	Man/Male	1080		17.1	
	Trans man/Male	781		12.4	
	Other	171		2.7	
	Trans woman/Female	143		2.3	
	Agender	115		1.8	
	Two-spirit	90		1.4	

^a^Pansexual, Panromantic, Pansensual.

^b^Bisexual, Biromantic.

^c^Asexual, Aromantic, Grey Asexual, etc.

^d^Demisexual, Demiromantic.

### Sample

Participants were age 14-29 (x bar=18.19, SD 3.60) and were able to select multiple options (or write in their own response) for both sexual orientation and gender identity. Responses were complex and individual. For simplicity, a multitude of related sexual orientations were grouped into five overarching “umbrella” categories (Table 2). Participants were also able to choose multiple racial and ethnic categories. The sample was predominantly Caucasian/white (79.57%, 5020/6309), though other categories also provided robust numbers: Hispanic (8.24%, 520/6309), mixed/multiracial (7.31%, 461/6309), American Indian/First Nations (5.31%, 335/6309), Asian (including South and Southeast Asian) (5.14%, 324/6309), Black (4.20%, 265/6309), and Middle Eastern (1.0105%, 66/6309).

 A third of the sample was from Canada (29.8399%, 1882/6309), while two thirds were from the United States (68.1516%, 4300/6309). A small number of participants came from outside these two countries (1.85%, 117/6309). Participants came from every state, province, and Canadian territory. The most American respondents came from California (7.35%, 316/4300), New York (5.00%, 215/4300), Ohio (4.79%, 206/4300), Florida (4.35%, 187/4300), Illinois (4.05%, 174/4300), Pennsylvania (4.00%, 171/4300), and Texas (3.65%, 157/4300). The most Canadian respondents came from Ontario (38.47%, 724/18821892), Alberta (16.47%, 310/18821892), British Columbia (16.26%, 306/18821892), and Quebec (7.07%, 133/18821892).

## Results

### Proposed Quantitative Analyses

Quantitative survey data have been entered into SPSS 24 and descriptive analyses conducted. Structural equation modeling (SEM) techniques will be used during analyses as they account for measurement error and error correlations, as well as interactions and latent variables [53]. AMOS 18.0 will be used to generate a SEM model of the multivariate relationships and evaluate it using recommended fit indices [54]. More focused SEM analyses will also be conducted by specific variables (eg, sexual orientation, gender and racialized identities; location; socioeconomic status) to identify commonalities and disparate patterns in ICT use, resilience, and coping.

### Proposed Qualitative Analyses

Qualitative videos and textual open-ended responses will be imported into ATLAS.ti 7 and analyzed using a grounded theory approach [55]. Video clips will be directly analyzed, as ATLAS.ti permits coding of video data. The use of video data has been lauded as a means of enabling participant voice in context [56,57], as an innovative method of triangulation [58], and as a technique to detect meaning and emotions not captured in text [59]. Open and axial coding strategies will be used to generate codes and themes that will subsequently be used to form a grounded theory to describe the relationship between ICTs and well-being for sexual and gender minority youth. To enhance trustworthiness, independent coders (research assistants) will be trained to perform thematic analysis and employ the “constant comparison” method [55].

### Proposed Integrative Analyses

A concurrent mixed methods analysis [60], using a data transformation design [61], will be used to effectively mine rich qualitative data while preserving the integrity of emergent qualitative themes. Multimethod research is ideal for resilience investigations [62] because such analytical integration addresses distinct multilevel protective processes (eg, individual, interpersonal, social, cultural) influencing resilience, resulting in more scientifically robust and culturally relevant theories [63]. This conceptual approach triangulates the data using investigator, theoretical, and methodological triangulation and creates a type of “ontological synthesis” that integrates an interwoven pattern of results into a comprehensive picture of interconnected phenomena [64].

Following full analysis of the qualitative findings, an integrative analytic process will be undertaken as follows:

1. The emergent themes developed from the grounded theory analysis will be quantitized (transforming qualitative data to a numerical form) to enhance the monomethod quantitative and qualitative results. For each participant’s textual response and/or video narrative, the presence or absence of each emergent theme of resilience and coping will be assigned a score of “1” or “0” [60].
2. Then an interrespondent matrix (ie, theme x youth) indicating which sexual and gender minority youth reflected each theme will be created [65].
3. A bivariate analysis of quantized responses (0 or 1) and selected demographic variables (eg, age, sexual orientation) will be conducted.
4. An exploratory factor analysis will determine constructs and results will generate metathemes (containing at least one emergent theme) that will contribute to a holistic theoretical understanding of resilience [66].

## Discussion

### Principal Considerations

Project #Queery will provide critical insights into sexual and gender minority youth's use of ICTs. It is the first study into the relationship between ICT use, mental health and health, and coping and risk behaviors of sexual and gender minority youth populations with participants from every state and province in the United States and Canada. This sample of English-speaking sexual and gender minority youth aged 14-29 (N=6309) describe their experiences and perceptions through quantitative (measures) and qualitative (text and video) data. With its innovative Web-based recruitment and survey design, the study encouraged significant involvement from sexual and gender minority youth who may not have interaction with offline services and may be less likely to participate in research [26]. Data collection is complete, with results of proposed analyses anticipated in 2017 and 2018.

### Strengths and Limitations

This project has several limitations, such as barriers to participation for sexual and gender minority youth who do not have access to ICTs or have literacy issues. That the sample is predominantly Caucasian/white (79.57%, 5020/6309) should also be acknowledged. In addition, this survey is fairly comprehensive and less accessible for sexual and gender minority youth who may not have had time to participate. Thus the results of this study will not be representative of all sexual and gender minority youth.

Despite these challenges, this project will develop an empirical understanding of the impact of ICTs on the well-being of sexual and gender minority youth, a socially vulnerable group who may be particularly active via ICTs [21]. Despite youth affinity for Web-based interventions [29,67,68], studies of their ICT use generally focus on problems (eg, online addiction, cyberbullying) [69,70] without adequate consideration of potential benefits. Online contexts are often welcoming spaces wherein youth may express themselves in ways not possible offline [71,72]. As sexual and gender minority youth frequently struggle with social exclusion and poor outcomes [9-15], understanding the potential of ICTs to minimize risks by promoting connections that help youth cope with and navigate their environment may significantly impact their lives [2]. This line of inquiry is critical to reducing the documented health and mental health disparities of sexual and gender minority youth. Emerging scholarship indicates sexual and gender minority youth benefit from ICTs [2,22-24,72] and supports our proposed analysis of the trends in use across a variety of subpopulations.

### Conclusion

Project #Queery will offer guidance for the development of best practices in ICT use for sexual and gender minority youth by offering insight into youth perceptions and the role of their social contexts. Knowledge mobilization will ensure that study findings are utilized by professionals, participants, and community. Presentations at relevant educational and social service settings, as well as professional and academic conferences, will be pursued. Findings will be disseminated through relevant academic journals and through the use of a series of 6-8 infographics to directly inform youth and practitioners. Results will be used to inform affirmative interventions to promote healthy coping of sexual and gender minority youth both online and offline. As ICT use is increasingly ubiquitous among youth [3-6], understanding the impact on sexual and gender minority youth is crucial to inform tailored intervention approaches.

## Appendix

Multimedia Appendix 1 Grant reviews.

## Abbreviations

ICT: information and communication technology
SEM: structural equation modeling
