# Supplementary material for: Influence of Information and Communication Technologies on the Resilience and Coping of Sexual and Gender Minority Youth in the United States and Canada (Project #Queery): Mixed Methods Survey
Source: JMIR Res Protoc. 2017 Sep 28;6(9):e189. doi: 10.2196/resprot.8397 (PMC5639209; doi:10.2196/resprot.8397)
Supplement: Multimedia Appendix 1 [file resprot_v6i9e189_app1.pdf]

SSHRC CRSH

350 Albert Street, P.O Box 1610
Ottawa ON K1P 6G4
www.sshrc.ca

350, rue Albert, C.P. 1610
Ottawa ON K1P 6G4
www.crsh.ca

Insight Grants

October 2014 Competition

Committee: 435-5F (Social Work)

File: 435-2015-0780

Applicant: Craig, Shelley L.

Application Title: Clicking to cope: the influence of information and communication technologies on the resilience, social support and school engagement of Canada's sexual and gender minority youth

---

Recommendation:

The committee recommended that this meritorious proposal be funded. At the same time, the committee considered that the proposed research could be accomplished with less than the amount requested. In particular, it found that savings could be made in the areas of website design and supplies.

Please consult the attached Notice of Award for the amount of your grant.

Scores:	Challenge	5.65 / 6
	Feasibility	5.55 / 6
	Capability	5.55 / 6
	Weighted Score	16.77 / 18
	Rank	4 / 38

Program Officer: Sue Geffken-Graham (613) 992-3407

SSHRC CRSH

Committee: 5F - Social Work
Applicant Name: Shelley Craig
Application Number: 435-2015-0780
Assessor Number: 2

Insight Grants

External Assessment Form

Assessment

Instructions: Evaluations by external assessors are intended to assist the committee in its deliberations. SSHRC is extremely grateful for your expertise as well as your time and effort.

Given the competitive nature of the adjudication process, constructive criticism and/or suggestions for improvement, if appropriate, may be helpful to the applicant.

As your assessment will be made available to the applicant, please do not include any personal identifying information. If such information appears in your document, the Council reserves the right to remove it.

Declarations on confidentiality and conflict of interest

a) The information provided in the applications is protected by Canada's *Privacy Act* and is made available to external assessors for reviewing purposes only. I therefore agree to treat as strictly confidential all the material from the above-mentioned file which has been submitted to me by the Council. After responding, be it positively or negatively, I will ensure the destruction of the said material.

Agree	X

b) I attest that I am not in a conflict of interest with the applicant(s).

Agree	X

Using the guidelines in the attached document, evaluate each sub-criteria below and check the appropriate box. Briefly explain your response.

Social Sciences and Humanities Research Council of Canada Conseil de recherches en sciences humaines du Canada
Canada
PROTECTED B WHEN COMPLETED / PROTÉGÉ B UNE FOIS REMPLI
Form/Formulaire 140

Committee: 5F - Social Work
Applicant Name: Shelley Craig
Application Number: 435-2015-0780
Assessor Number: 2

Part 1: Challenge — The aim and importance of the endeavour

Sub-criteria (No specific weighting assigned to each sub-criterion)	N/A	Modest	Good	Very Good	Excellent
a) Originality, significance and expected contribution to knowledge					X
b) Appropriateness of the literature review				X	
c) Appropriateness of the theoretical approach or framework				X	
d) Appropriateness of the methods/approach				X	
e) Quality of training and mentoring to be provided to students, emerging scholars and other highly qualified personnel, and opportunities for them to contribute					X
f) Potential influence and impact within and/or beyond the social sciences and humanities research community					X

g) Briefly describe the proposal's strengths and weaknesses in relation to the above criteria:

This is timely and important research. As the applicants point out, while there is much research and publicity surrounding the adverse and potential dangers of ICTs it's potential and real uses and benefits to society remain muted, under-studied, and ignored. The proposed study extends and develops an important first step in understanding the important ways in which ICTs can be used to promote positive outcomes for youth. The theoretical framework in which the study is situated ensures that findings can be understood within a broader continuum of knowledge. In this way, the study also stands to be of great relevance beyond both the SGMY community and resilience and school engagement outcomes. As an example, findings could be of great relevance to supporting positive mental health outcomes for Aboriginal youth living in rural and remote communities of Canada. Furthermore, the ways in which students and community members are integrated into the design ensures excellent opportunities for training and mentoring.

Part 2: Feasibility — The plan to achieve excellence

Sub-criteria (No specific weighting assigned to each sub-criterion)	N/A	Modest	Good	Very Good	Excellent
a) Probability of effective and timely attainment of the research objectives					X
b) Appropriateness of the requested budget, and justification of proposed costs				X	
c) Indications of financial and in-kind contributions from other sources, where appropriate				X	

Committee: 5F - Social Work
Applicant Name: Shelley Craig
Application Number: 435-2015-0780
Assessor Number: 2

Sub-criteria (No specific weighting assigned to each sub-criterion)	N/A	Modest	Good	Very Good	Excellent
d)Quality of knowledge mobilization plans, including for effective knowledge dissemination, knowledge exchange and engagement within and/or beyond the research community				X	
e) Strategies and timelines for the design and conduct of the activity/activities proposed					X

f) Briefly describe the proposal's strengths and weaknesses in relation to the above criteria:

The proposed plan seems extremely feasible both in terms of research and knowledge mobilisation. The timelines are achievable and the activities well supported by the budget. Knowledge mobilisation activities are well balanced and will ensure dissemination to a varied and relevant audience.

Part 3: Capability — The expertise to succeed

Please note that in the case of a research team, you will need to evaluate the strength and suitability of the team members' research achievements (do not include collaborators).

In your evaluation of this scholar's or team's capability to succeed, address the following criteria while **considering his/her career stage:**

Sub-criteria (No specific weight assigned to each sub-criterion)	N/A	Modest	Good	Very Good	Excellent
a) Quality, quantity and significance of past experience and published outputs of the applicant and any team members relative to their roles in the project and their respective stages of career					X
b) Evidence of contributions such as commissioned reports, professional practice, public discourse, public policies, products and services, development of talent, experience in collaboration, etc.				X	
c) Potential to make future contributions				X	

d) Briefly describe the proposal's strengths and weaknesses in relation to the above criteria:

The applicant and co-applicants have a solid track record and are well positioned to conduct a strong study, with quality outputs that are sure to inform future research, related work, and policy across disciplines, regions and populations.

Part 4: Additional Comments

Committee: 5F - Social Work
Applicant Name: Shelley Craig
Application Number: 435-2015-0780
Assessor Number: 2

a) If you have comments regarding the budget or other aspects of the proposal, please include them here:
